# Differential Expression and miRNA–Gene Interactions in Early and Late Mild Cognitive Impairment

**DOI:** 10.3390/biology9090251

**Published:** 2020-08-28

**Authors:** Leonardo Miranda Brito, Ândrea Ribeiro-dos-Santos, Amanda Ferreira Vidal, Gilderlanio Santana de Araújo

**Affiliations:** 1 Laboratório de Genética Humana e Médica, Instituto de Ciêncas Biológicas, Universidade Federal do Pará, Belém 66075-110, Brazil; lb9458@gmail.com (L.M.B.); akelyufpa@gmail.com (Â.R.-d.-S.); amandaferreiravidal@gmail.com (A.F.V.); 2 Programa de Pós-Graduação em Genética e Biologia Molecular, Instituto de Ciêncas Biológicas, Universidade Federal do Pará, Belém 66075-110, Brazil

**Keywords:** mild cognitive impairment, Alzheimer’s Disease, genes, miRNAs, regulatory interactions

## Abstract

Mild cognitive impairment (MCI) and Alzheimer’s Disease (AD) are complex diseases with their molecular architecture not elucidated. *APOE*, Amyloid Beta Precursor Protein (*APP*), and Presenilin-1 (*PSEN1*) are well-known genes associated with both MCI and AD. Recently, epigenetic alterations and dysregulated regulatory elements, such as microRNAs (miRNAs), have been reported associated with neurodegeneration. In this study, differential expression analysis (DEA) was performed for genes and miRNAs based on microarray and RNA-Seq data. Global gene profile of healthy individuals, early and late mild cognitive impairment (EMCI and LMCI, respectively), and AD was obtained from ADNI Cohort. miRNA global profile of healthy individuals and AD patients was extracted from public RNA-Seq data. DEA performed with *limma* package on ADNI Cohort data highlighted eight differential expressed (DE) genes (*AGER*, *LINC00483*, *MMP19*, *CATSPER1*, *ARFGAP1*, *GPER1*, *PHLPP2*, *TRPM2*) (false discovery rate (FDR) *p*-value < 0.05) between EMCI and LMCI patients. Previous molecular studies showed associations between these genes with dementia and neurological-related pathways. Five dysregulated miRNAs were identified by DEA performed with RNA-Seq data and *edgeR* (FDR *p*-value < 0.002). All reported miRNAs in AD interact with the aforementioned genes. Our integrative transcriptomic analysis was able to identify a set of miRNA–gene interactions that may be involved in cognitive and neurodegeneration processes.

## 1. Introduction

Alzheimer’s Disease (AD) is a multifactorial and heterogeneous genetic disorder. Early-onset patients carry mutations or polymorphisms in multiple genes, such as Amyloid Beta Precursor Protein (*APP*), Presenilin-1 (*PSEN1*) and Presenilin-2 (*PSEN2*) [1]. In contrast, late-onset patients generally carry *APOE* isoforms [2,3,4]. Preceding severe AD, some patients manifest mild cognitive impairment (MCI), which is a syndrome that involves cognitive decline without interference in the performance of individuals’ daily tasks [5]. MCI is a clinical stage between normality and dementia, and recently was divided into two stages, early MCI (EMCI) and late (LMCI) [6,7]. From the genetic perspective, *APOE* remains as the most statistically associated gene to MCI, mainly ϵ4 allele [3,8]. Gene expression profiling of MCI patients is more similar to healthy individuals than with AD patients [9].

Gene profiling improved our knowledge and constantly provides insights about the molecular structure of neurodegeneration and other diseases. Moreover, only gene expression analysis is lacking to effectively explain MCI and AD development process, prognostic, or diagnostic. Epigenetic alterations have also been associated with AD, including DNA methylation, histone modifications, and aberrant expression of non-coding RNAs (ncRNAs) [10]. microRNAs (miRNAs) are the most well-known class of ncRNAs—their regulatory function leads to translation repression and target mRNA degradation [11]. Several miRNAs have been identified to directly regulate AD-associated genes, and some studies showed that the interaction between AD-associated polymorphisms and miRNA gene regulation may affect AD susceptibility [10,12].

**Figure 1:**
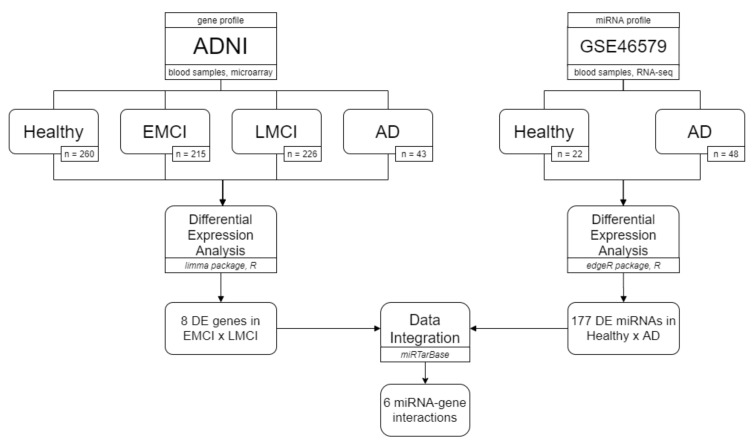
Overview of experimental data, statistical analysis, databases, differential expression analysis (DEA) results, and data integration.

To identify dysregulated molecular elements, we systematically analyzed the mRNA profile of healthy individuals, early MCI, late MCI, and AD patients from ADNI cohort [13], and also the global miRNA expression profile (miRNome) of AD patients from public datasets [12]. Differential expression analysis (DEA) aims to highlight quantitative changes in genes or miRNAs expression, between experimental profiles, i.e., healthy × case. Dysregulated genes or miRNAs identified by DEA are hereafter named as DE genes and DE miRNAs, respectively. We proposed to highlight regulatory interactions between DE genes and DE miRNAs in MCI and severe dementia. Our miRNA–gene interactions comprise a potential set of regulatory elements and genes that may regulate the progression of cognitive decline and neurodegeneration and are potentially useful as early diagnostic biomarkers and as therapeutic targets. A summary of our data, statistical analysis, software, and results are represented in Figure 1, which is detailed in the next sections.

## 2. Materials and Methods

### 2.1. Gene Expression Data

Data used in the preparation of this article were obtained from the ADNI database (https://adni.loni.usc.edu). The ADNI was launched in 2003 as a public–private partnership, led by Principal Investigator Michael W. Weiner, MD. The primary goal of ADNI has been to test whether serial magnetic resonance imaging (MRI), positron emission tomography (PET), other biological markers, and clinical and neuropsychological assessment can be combined to measure the progression of mild cognitive impairment (MCI) and early Alzheimer’s disease (AD). The Principal Investigator of this initiative is Michael W. Weiner, MD, VA Medical Center and University of California, San Francisco. ADNI is a global research effort that actively supports the investigation and development of treatments that slow or stop the progression of AD and subjects have been recruited from over 50 sites across the US and Canada. The overall goal of ADNI is to determine biomarkers for use in Alzheimer’s disease clinical treatment trials. To date, it has three phases: ADNI-1, ADNI-GO and ADNI-2, consisting of cognitively normal (CN) individuals, early mild cognitive impairment (EMCI), to late mild cognitive impairment (LMCI), and dementia or AD. For more information, see http://www.adni-info.org. Institutional review board approval was conducted at each ADNI site. Written informed consent was obtained from all participants or authorized representative.

On ADNI-2 phase, microarray-based RNA gene expression profiles were generated for 260 controls, 451 with MCI being 215 EMCI and 226 LMCI patients and 43 AD patients, using the Affymetrix Human Genome U219 Array (Affymetrix, Santa Clara, CA, USA). The microarray captured expression for 49,293 transcripts related to 20,093 genes. All eligible subjects passed by an inclusion/exclusion criteria regarding the details at http://www.adni-info.org.

Additionally, public gene expression data was accessed for brain tissue analysis from the Genotype-Tissue Expression Portal (GTEx) (https://gtexportal.org/home/) [14], and RNA-Seq data from Aging, Dementia and Traumatic Brain Injury Study by its portal available in (http://aging.brain-map.org/) [15].

### 2.2. MicroRNA Expression Data

Global miRNA expression data (miRNome) were generated by Leidinger et al. (2013) [12] and downloaded from Gene Expression Omnibus with accession number GSE46579. The miRNome was extracted from total RNA from blood samples of AD patients and healthy individuals (control group). Samples were sequenced for small RNA-Seq using Illumina HiSeq 2000. AD patients are composed of 23 males and 25 females (mean of age = 72.7 (+/−10.4) and a mean of Mini-Mental State Examination (MMSE) equals 18.9 (+/−3.4). Healthy individuals are 11 males and 11 females, with a mean of age 67.1 (+/−7.5) and a mean of MMSE equal to 29.3 (+/−1.2). Overall, the miRNA data comprise 503 miRNAs expressed in both groups.

### 2.3. Differential Expression Analysis of Gene and miRNA

Gene expression profile was measured for healthy individuals, EMCI, LMCI, and AD. These data were stored in ADNI database. DEA were performed using *limma* library in a R environment (v3.5) [16]. False discovery rate (FDR) was used for multiple test correction, and those genes with adjusted *p*-value < 0.05 were considered statistically significant. We performed two DEA of genes for ADNI groups, being the first one at probe set level and the second considering the mean of each probe set analysis to avoid variation on gene expression, since probes for the same transcript may show expression variation regarding its affinity or position on the microarray chip.

For miRNome analysis, DEA were performed with *egdeR* package based on public pre-processed and normalized counts provided under GEO accession GSE46579 [17]. Graphic analysis was plotted using *ggplot2* and *ComplexHeatmap* in R environment (v3.5) [18,19]. Validated target genes of the DE miRNAs were extracted from miRTarBase database (Release 7.0: 15 September 2017), considering only those that were validated by strong evidences (reporter assay, western blot and qPCR) [20].

## 3. Results

Global gene expression profile of controls, EMCI, LMCI, and AD patients from the ADNI cohort were tested for differential expression, as well as an independent miRNome profile of healthy individuals and AD patients. In a pairwise analysis, we performed DEA aiming to find a set of genes and miRNAs with abnormal expression in cognitive decline and Alzheimer’s Disease.

### 3.1. Differential Expression in EMCI and LMCI Gene Profile

**Figure 2:**
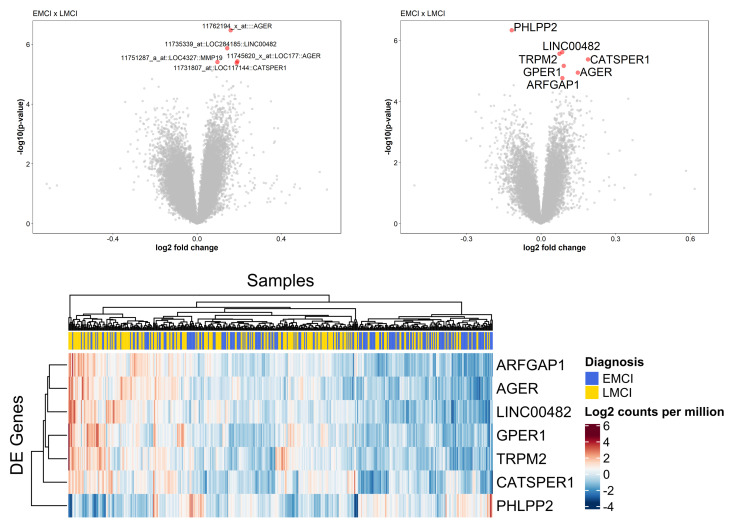
On top, volcano plots of DEA results. On the top left, DEA results for genes in probe expression. On the top right, DE genes at the mean of probe set expression. In total, eight genes were found to be differentially expressed in EMCI and LMCI. *AGER*, *LINC00482*, *MMP19*, *CATSPER1*, *PHLPP2*, TRPM2, *ARFGAP1*, *GPER1* genes are in red with FDR *p*-value < 0.05. On the bottom, heatmap and hierarchical clustering of DE genes in EMCI and LMCI.

DEA for genes was performed for all pairwise groups of ADNI cohort. Among the 49K probes, we found five DE probes related to four genes (Advanced Glycosylation End-Product Specific Receptor—*AGER*, Long Intergenic Non-protein Coding RNA 483—*LINC00483*, Matrix Metallopeptidase 19—*MMP19*, and Cation Channel Sperm Associated 1—*CATSPER1*), concerning EMCI and LMCI experimental gene profile (Table 1). We also performed a second round of DEA regarding the mean of probe set levels, which pointed seven DE genes, being three in intersection with the first comparison (PH Domain And Leucine Rich Repeat Protein Phosphatase 2—*PHLPP2*, Transient Receptor Potential Cation Channel Subfamily M Member 2—*TRPM2*, G Protein-Coupled Estrogen Receptor 1—*GPER1*, *AGER* and ADP Ribosylation Factor GTPase Activating Protein 1—*ARFGAP1*, *LINC00482*, *CATSPER1*). Among all of these DE genes, seven genes were up-regulated (*AGER, LINC00482, MMP19, CATSPER1, TRPM2, GPER1, ARFGAP1*) and only one showed down-regulated (*PHLPP2*) in LMCI patients in comparison to EMCI. Statistical results and genomic annotations for DEA are summarized in Table 1. DE genes were graphically represented in volcano plots and heatmaps (see Figure 2). These findings suggest that LMCI and EMCI substages do not have massive gene expression differences. Due to microarray technologies, we suggest new investigations with RNA-Seq data to confirm these results.

**Figure 3:**
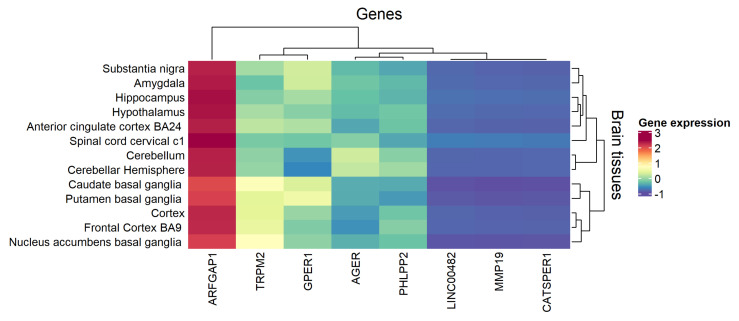
Heatmap of median gene expression for DE genes in brain tissues. Data were available in GTEx portal (https://www.gtexportal.org/home/).

Transcriptome summary data from GTEx allowed us to analyze their expression among different brain regions, based on RNA-Seq data. Overall, the most expressed gene was *ARFGAP1*, and particularly the *CATSPER1*, *MMP19*, and *LINC00482* is grouped as less expressed genes in brain regions (see Figure 3).

### 3.2. Differential Expression on miRNome and miRNA–Gene Interactions

We performed DEA on AD miRNome and found 177/503 dysregulated miRNAs (Supplementary Table S1). Altogether, these 177 DE miRNAs can regulate 890 target genes (Supplementary Table S2). We merged results of DEA for genes and miRNAs with miRTarBase interaction data, and then filter to focus only interactions between DE genes in EMCI and LMCI and DE miRNAs in AD. We identified six miRNA–gene interactions with experimental studies regarding AD or AD-related processes, such as inflammation and oxidative stress (Table 2). Three down-regulated miRNA interacts with AGER and *GPER1*, and two up-regulated miRNAs interact with *PHLPP2* and *LINC00483*.

## 4. Discussion

In this study, we explored the gene expression profile regarding healthy individuals, EMCI, LMCI, and AD patients, and the miRNA profile in controls and AD patients aiming to find a set of genes and miRNAs with abnormal expression in these neurological conditions. We found eight DE genes, such as the *AGER*, *MMP19*, *GPER1*, and *TRPM2*, were previously associated with neurodegeneration processes and related pathways [27,28,29,30,31,32,33,34,35,36].

*AGER* (also known as *RAGE*) is deeply related to the progress of AD through its influence in the inflammatory pathway, oxidative stress induction, beta-amyloid production, and accumulation, failure of synaptic transmission, and neuronal degeneration [27,28,29]. In addition, *AGER* may increase the risk of cognitive decline in diabetic patients as an age-induced MCI factor [37].

The matrix metalloproteinases family has been associated with neuroinflammation, progress of neurodegenerative diseases, and brain cancer, including MCI and AD [31,38,39]. For instance, MMP9 was found overexpressed in MCI and AD. Its activity was found inversely correlated with cognitive scores, such as Global Cognitive Score and Mini-Mental State Examination [40]. Our study identified overexpression of MMP19 that was previously associated with cerebral amyloid angiopathy, which is one of the active processes commonly found in the AD progress [32]. Interestingly, MMP19 was found expressed in the white matter of healthy individuals, especially in microglia throughout the brain parenchyma [41]. These findings reinforce that microglia activation may be an important process for the aetiopathogenesis of mild cognitive impairment, leading to the progress of Alzheimer’s syndrome [42].

Our DEA results show that *GPER1* is overexpressed in LMCI. In addition, we checked GPER1 tendency of overexpression in RNA-Seq data from the Aging, Dementia, and Traumatic Brain Injury Study (http://aging.brain-map.org/), which confirmed its overexpression in dementia patients in comparison with healthy individuals (Wilcox Test *p*-value = 6.0 × 10${}^{-8}$). Agonists of GPER1, such as 1-[4-(6-bromobenzo[1,3] dioxol-5yl)-3a,4,5,9b-tetrahydro-3H-cyclopenta[c]quinolin-8-yl]-ethanone (G-1) have been tested as an anticancer by suppressing cancer cell proliferation [30]. Recently, an experimental study conducted with rat cortex cells tested the effect in different situations of neurotoxicity. The authors showed that G-1 increased cell survival, which suggests that early activation of *GPER1* may improve therapeutics in neurodegeneration [43].

*TRPM2* is also related to MCI and AD, and its aberrant expression is associated with oxidative stress that can lead to aberrant calcium intracellular concentration and cell death [33,34]. We also used the Aging, Dementia, and Traumatic Brain Injury Study (http://aging.brain-map.org/) dataset, following the same criteria of comparison described for *GPER1*, and also found *TRPM2* overexpression in dementia patients (Wilcoxon test *p*-value = 6.63 × 10${}^{-6}$).

Additionally, we highlight *PHLPP2*, *ARFGAP1*, *CATSPER1*, and *LINC00482* as four novels DE genes, not previously described in progress or protection of developing neuronal tissue disorders. *LINC00482* is a long intergenic non-coding RNA (lincRNA), also known as *C17orf55*, located at chromosome 17. No functional information about this gene is available. However, it is important to note that the brain presents a remarkable abundance of tissue-specific ncRNAs, including lincRNAs, which reinforce the need for further studies to reveal the biological roles of *LINC00482* [35].

Among all DE miRNAs, five of them were previously associated with AD and also target dysregulated genes in MCI (Table 2). A study made in a transgenic mouse model of Alzheimer’s Disease (Heterozygous APPswe/PS1Δ9 transgenic founder mice) showed that *mmu-miR-10a-5p* is up-regulated in these transgenic mice when compared with wild mice, in *miR-10a-5p* is conserved between both human and mouse brain [44]. In addition to that, *hsa-miR-10a-5p* has been associated with regulation of exocytosis transmission, in a co-expression network study [45], and to Parkinson’s Disease, in an miRNA profile analyses from cerebrospinal fluid exosome [36].

Both *hsa-miR-151a-5p* and *hsa-miR-151b* interact with *GPER1*, but only the first was reported previously as potential blood biomarkers of AD [22,46]. The latter is associated with a neurodegenerative disease called manganism [47], which causes motor deficits similar to those of Parkinson’s Disease [48].

In our study, *hsa-miR-15a-5p* is up-regulated and interacts with *PHLPP2* gene. Meta-analysis studies reported *hsa-miR-15a-5p* related to apoptosis in severe dementia [49]. In addition, *hsa-miR-15a* was found improving dementia process in animal models. Downregulation of *hsa-miR-15a* in the AD mouse brain may cause unbalances in tau phosphorylation [50].

Tissue-specific expression of *hsa-miR-26b* shows an antagonistic dysregulation behavior. While it is down-regulated in the temporal cortex, it was up-regulated in the blood of AD samples. *hsa-miR-26b* was observed overexpressed in MCI and AD patients with increased expression levels in the MCI stage and remains elevated in some pathological areas of the AD brain [51]. Its up-regulation found in the brain impacts neuronal cells increasing tau phosphorylation, which leads to apoptotic cell death.

Recently, some reports have associated lysosomal and autophagic dysfunction with AD, since it leads to a deficit in clearance of beta-amyloid and accumulation of amyloid precursor protein metabolites [52]. Interestingly, *PHLPP2*, which is target of *hsa-miR-15a-5p* and *hsa-miR-26b-5p* (Table 2), is involved in the control of autophagy in cancer [53], suggesting that this gene may also be related to autophagic dysfunction in the brain.

In summary, we found four genes previously associated with AD, as well as another four that are unprecedented in the context of neurodegeneration. Out of the total eight DE genes, four are linked to five DE miRNAs. All of these miRNAs are related to neurodegeneration processes.

## 5. Conclusions

In this study, we integrated transcriptomic data of MCI and AD, and found a set of DE genes that are potentially involved in cognitive decline and neurodegenerative diseases. Among the set of genes, we highlight some well-known AD-related genes, but also some novel genes, such as *PHLPP2, ARFGAP1*, and *CATSPER1*. The miRNA–gene interactions described in the present study are a valuable set of regulatory elements that must be used as potential candidates as biomarkers of cognitive and severe dementia, however, it requires more functional studies to validate these interactions. Our future work includes validating these findings with gene profiles based on RNA-seq data as well as performing co-expression analysis to investigate regulatory module on networks between miRNAs and genes.

## Figures and Tables

**Table 1:** DEA statistical results for probes gene level and DEA for the mean of probe set expression level for early mild cognitive impairment (EMCI) versus late mild cognitive impairment (LMCI). Chr: chromosome; LogFC: log2-fold-change; AveExpr: average expression; T: Student’s t-test value; FDR: false discovery rate.

Probe Expression Level						
**Gene**	**Chr**	**Region**	**LogFC**	**AveExpr**	***p*** **-Value**	**FDR (** ***p*** **-Value)**
*AGER* (11762194_x_at)	6	6p21.32	0.16	4.86	3.38 × 10${}^{-7}$	0.016
*LINC00482* (11735339_at)	17	17q25.3	0.14	3.80	1.35 × 10${}^{-6}$	0.033
*AGER* (11745620_x_at)	6	6p21.32	0.19	5.80	3.68 × 10${}^{-6}$	0.041
*MMP19* (11751287_a_at)	12	12q13.2	0.09	2.44	3.89 × 10${}^{-6}$	0.041
*CATSPER1* (11731807_at)	11	11q13.1	0.18	6.31	4.16 × 10${}^{-6}$	0.041
**Mean of Probe Expression Level**						
**Gene**	**Chr**	**Region**	**LogFC**	**AveExpr**	***p*** **-value**	**FDR (** ***p*** **-value)**
*PHLPP2*	16	16q22.2	−0.1184	3.60	4.62 × 10${}^{-7}$	0.009
*LINC00482*	17	17q25.3	0.0851	3.05	2.39 × 10${}^{-6}$	0.018
*TRPM2*	21	21q22.3	0.0740	3.33	2.70 × 10${}^{-6}$	0.018
*CATSPER1*	11	11q13.1	0.1888	6.31	4.14 × 10${}^{-6}$	0.020
*GPER1*	7	7p22.3	0.0911	3.68	6.74 × 10${}^{-6}$	0.027
*AGER*	6	6p21.32	0.1478	5.76	1.13 × 10${}^{-5}$	0.037
*ARFGAP1*	20	20q13.33	0.0855	4.79	1.70 × 10${}^{-5}$	0.048

**Table 2:** Differentially expressed miRNAs in Alzheimer’s Disease (AD) targets differentially expressed genes in MCI.

miRNA	logFC	FDR (*p*-Value)	Experiment	Target Gene	References
hsa-miR-10a-5p	−0.699380073	5.8 × 10${}^{-3}$	CLASH	*AGER*	[21]
hsa-miR-151a-5p	−0.345619322	3.4 × 10${}^{-2}$	HITS-CLIP	*GPER1*	[22]
hsa-miR-151b	−0.345583534	3.4 × 10${}^{-2}$	HITS-CLIP	*GPER1*	[23]
hsa-miR-15a-5p	1.292271145	3.9 × 10${}^{-7}$	PAR-CLIP	*PHLPP2*	[24,25]
hsa-miR-26b-5p	0.692598872	2.1 × 10${}^{-3}$	Microarray	*PHLPP2*	[12,26]
hsa-miR-26b-5p	0.692598872	2.1 × 10${}^{-3}$	Microarray	*LINC00483*	[12,26]

## Supplementary Materials

The following are available online at https://www.mdpi.com/2079-7737/9/9/251/s1, Table S1: List of DE miRNAs, Table S2: List of miRNA-gene interactions. Data used in preparation of this article were obtained from the Alzheimer’s Disease Neuroimaging Initiative (ADNI) database (adni.loni.ucla.edu). As such, the investigators within the ADNI contributed to the design and implementation of ADNI and/or provided data, but did not participate in analysis or writing of this report. A complete listing of ADNI investigators can be found at: http://adni.loni.ucla.edu/wp-content/uploads/how_to_apply/ADNI_Acknowledgement_List.pdf.

## Abbreviations

The following abbreviations are used in this manuscript:

AD	Alzheimer’s Disease
EMCI	Early Mild Cognitive Impairment
LMCI	Late Mild Cognitive Impairment
MMSE	Mini-Mental State Exam
